# Insulinoma Mimicking Psychiatric Illness: A Covert Endocrine Tumor

**DOI:** 10.7759/cureus.33788

**Published:** 2023-01-15

**Authors:** Sidra Aslam, Ahmed I Siddiqi, Waqas Shafiq, Umal Azmat, Hira Irfan, Wania Rafaey, Sidra Masood

**Affiliations:** 1 Endocrinology and Diabetes, Shaukat Khanum Memorial Cancer Hospital and Research Centre, Lahore, PAK; 2 Internal Medicine, Liaquat National Hospital, Karachi, PAK

**Keywords:** pancreas, hyperinsulinemia, neuroendocrine tumor, hypoglycemia, insulinoma

## Abstract

Insulinomas are rare neuroendocrine tumors with an annual incidence of four cases per million people in the general population. They have varied presentations making their diagnosis a challenging task necessitating a thorough patient assessment to ascertain early detection of this clinical entity by treating physicians. Insulinomas are characterized by the presence of Whipple's triad comprising of hypoglycemic symptoms, biochemical demonstration of hypoglycemia, and improvement of those symptoms after glucose administration. Biochemical detection of insulinoma by supervised 72-hour fasting test with plasma glucose, insulin, C-peptide, and proinsulin level measurements remains the gold standard of diagnosis. In this report, we present an interesting case of delayed diagnosis of pancreatic insulinoma. He was treated for more than six years as a psychiatric illness before receiving the correct diagnosis and treatment. Herein, a middle-aged man with a history of recurrent episodes of altered talk and confusion that resolved after eating something sweet. Biochemical investigations were suggestive of endogenous hyperinsulinemia. Pancreatic insulinoma was localized by a computed tomography scan. The patient underwent surgical resection of the tumor with complete resolution of his symptoms.

## Introduction

Insulinomas are the commonest neuroendocrine tumors of pancreatic origin. This condition is a diagnostic dilemma as it is rare and often presents with non-specific symptoms. Patients with insulinoma do not undergo diagnostic investigations in time, and optimal treatment is delayed by a mean of 3.8 years [1]. Insulinomas often present in the fifth decade; however, an earlier presentation is seen in patients with multiple endocrine neoplasia (MEN-1), typically during the second decade of life [2]. Insulinomas are typically located within the pancreas; however, extra-pancreatic insulinomas have been recognized. Insulinomas present with neuroglycopenic symptoms that may or may not be accompanied by autonomic symptoms [3].

## Case presentation

**Figure 1:**
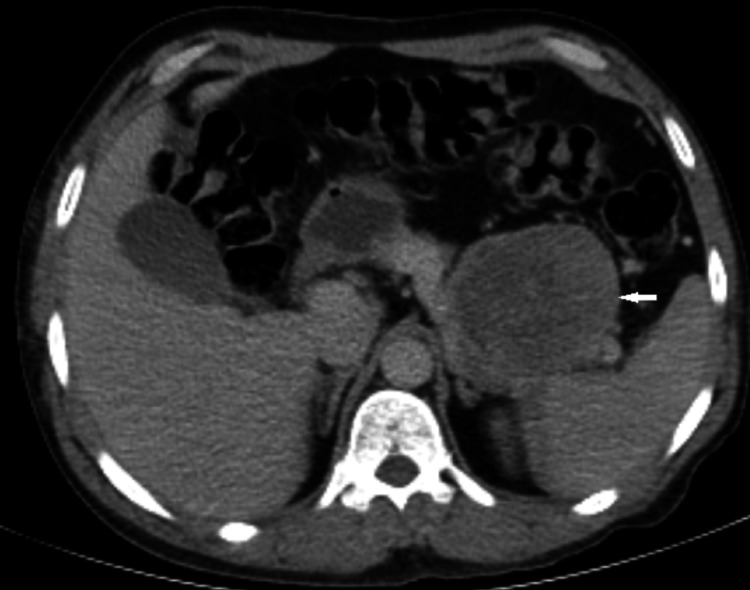
Pre-operative axial CT section at upper abdomen showing an enhancing mass in the tail of the pancreas

A 47-year-old man with a history of recurrent episodes of altered level of consciousness for the last six years that involved periods of confused talk and behavior attended the endocrine clinic. The patient's brother mentioned that his symptoms improved with sweetened products and sugary drinks. Initially, he was diagnosed with epilepsy and started on carbamazepine. Later, when his paranoid behavior and abusive language continued, he was started on antipsychotics, which he took for almost six years without any improvement in symptoms. The patient had a recent admission to a local hospital with profuse vomiting, where he transiently developed a depressed level of consciousness. A note was made of low blood glucose, which was recorded twice during admission that required immediate correction with 25% dextrose water injections. His further workup included a computed tomography scan of the abdomen (Figure 1) followed by endoscopic ultrasound-guided FNAB (fine needle aspiration biopsy) of pancreatic masses, which led to a clinical diagnosis of neuroendocrine tumor. The patient's family denied the use of any hypoglycemic drugs. Upon presentation to the endocrine clinic, he was afebrile, normotensive, pulse rate of 72/min, and had a respiratory rate of 14/min. The patient had a body mass index of 25.06 kg/m with a normal neurological and cardiorespiratory examination. His brother mentioned that the patient was having difficulty recalling the details of the recent past. He was admitted for 72-hour prolonged fasting, and biochemical investigations for insulinoma were carried out (Table 1).

**Table 1:** Laboratory investigations TSH - thyroid stimulating hormone

Variable	Unit	Reference range
Insulin	34 μIU/ml	Adult (fasting) 2.5- 25
Glucose (fasting)	23 mg/dL	70-99
C-peptide	3.9 ng/mL	0.9-7.1
TSH	1.4 µIU/mL	0.35-5.5
Cortisol 9am	13.8 µg/dL	4.30-22.4
Calcium corrected	9 mg/dl	8.5 - 10.5
Human growth hormone	3.37 ng/mL	0-3

**Figure 2:**
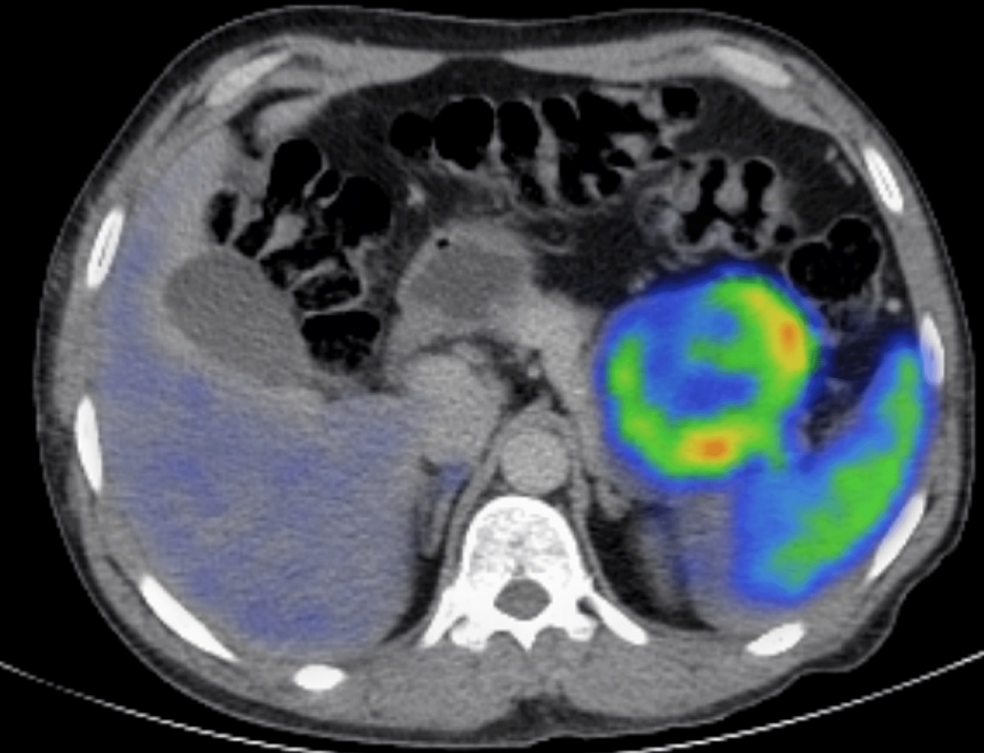
Ga-68 DOTA PET/CT pre-operative image showing pancreatic mass with increased uptake DOTA - dodecane tetraacetic acid, PET - positron emission tomography

Given the aforementioned biochemical investigations and imaging, Ga-68 dodecane tetraacetic acid (DOTA) positron emission tomography (PET)/CT was performed to rule out any other foci (Figure 2), which was diagnostic of a neuroendocrine tumor. It demonstrated two DOTA avid masses located at the distal part of the pancreas measuring 7.5 x 6.6 cm with standardized uptake values (SUV) of 35.3 and another one inferior to the spleen measuring 7.1cm x 6.1 cm with SUV of 40.3. No distant metastases were identified.

**Figure 3:**
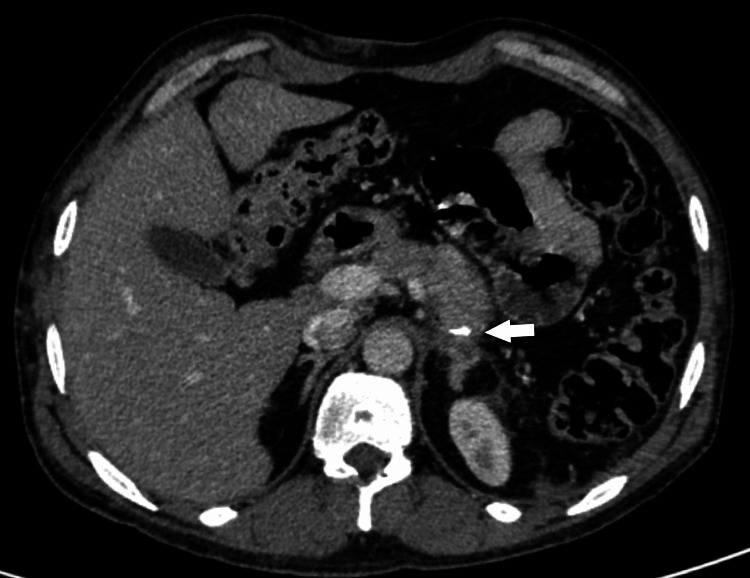
Axial CT showing post-operative changes with visible surgical staples

**Figure 4:**
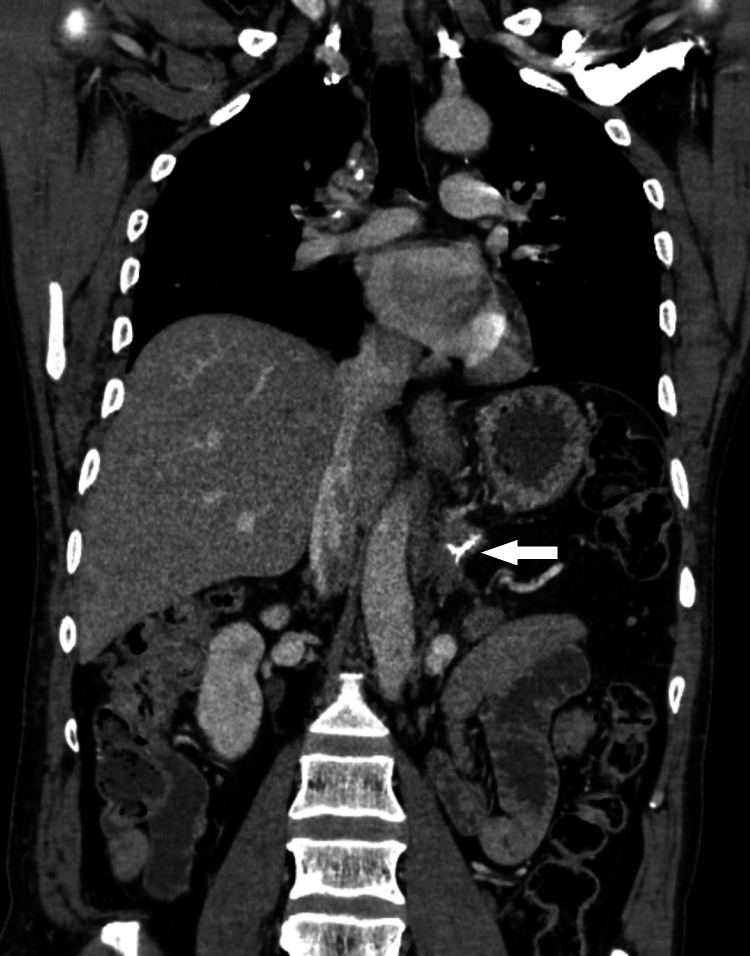
CT chest/abdomen coronal image showing post-operative changes with no evidence of recurrence

The findings were discussed in a multidisciplinary team meeting and planned for surgical resection of the tumor. The patient underwent laparoscopic distal pancreatectomy and splenectomy. Histopathology report showed a 130mm, unifocal tumor found in the tail of the pancreas, limited to the pancreas with clear margins. The report showed a neuroendocrine tumor, WHO grade 2, with Ki67: 5% proliferation index and positive synaptophysin. The patient was followed up in the clinic after three months with both biochemical investigations and imaging (Figures 3, 4), neither of which showed any evidence of recurrence. The patient initially required insulin administration for a couple of months which was later stopped.

## Discussion

An Insulinoma presents with symptoms of hypoglycemia occurring secondary to excessive production of insulin by a tumor of pancreatic beta-cells [4]. When insulinoma presents with altered behavior, it may be misinterpreted as a psychiatric illness. Symptoms may range from fatigue, confusion, poor concentration, irritability, and impaired memory to seizures and loss of consciousness" [5]. The presentation of hypoglycemia with psychiatric symptoms is well documented in the literature. However, in our patient it took a lot longer before he was diagnosed with insulinoma.

Insulinomas mostly follow a benign course, presenting as a solitary lesion with a size of <2cm diameter, whereas multiple lesions occur in 5-10% of cases and are mostly associated with multiple endocrine neoplasia (MEN) 1 or 4 [6]. Our patient had the tumor for so many years and the tumor got a lot bigger in size. Laboratory workup for our patient excluded MEN syndrome. A suitable diagnostic test for insulinoma is a 72-hour supervised fasting test showing inappropriate elevation of insulin (≥6 μU/mL) and C-peptide (≥0.2 nmol/L or >0.6ng/ml) levels in the presence of hypoglycemia (<54mg/dl ) [3].

Imaging techniques to detect the tumor include both noninvasive techniques such as transabdominal ultrasound, computed tomography (CT), magnetic resonance imaging (MRI), Ga-68 DOTA PET/CT scan, and also invasive such as endoscopic ultrasound, which may or may not be accompanied by biopsy. They have varying detection rates, with a sensitivity of CT and MRI approaching 70-85% [7]. 68Ga-DOTA PET/CT has been reported to localize neuroendocrine tumors with a size as small as 6mm with an additional advantage to exclude the presence of additional pancreatic neuroendocrine tumors not detected by anatomic imaging in inherited syndromes such as MEN1 [8]. Our patient had a multifocal lesion on CT abdomen; therefore, in order to exclude any other foci, we carried out a Ga-68 DOTA PET/CT scan.

Surgery is the treatment of choice in a patient with insulinoma. Early treatment can prevent potential neurological damage associated with prolonged hypoglycemia [9]. Our patient had a history of memory loss; however, no clinically detectable hypoglycemic brain damage was noted despite so many years of undiagnosed potentially prolonged episodes of hypoglycemia. No functional scans to assess brain damage were done on our patient. On his post-operative follow-up at three months, his Mini-Mental State Examination (MMSE) score was 26/30, and he was able to function independently without requiring family support.

## Conclusions

We would like to emphasize that clinicians should screen for insulinoma when a patient present with neuroglycopenic symptoms before diagnosing a patient with psychiatric illness. Insulinomas are notorious for being exceptionally small and challenging to diagnose. However, sometimes size is not to be blamed, as in our case. Eyes do not see what the mind does not know. A high index of suspicion would be the key to not missing this rare but important diagnosis. Insulinomas can be treated effectively with surgery, and early localization and treatment can prevent serious adverse effects, including neurological damage.
